# Opposing Influences of Optic Neuritis and Transverse Myelitis on the Future Location of Relapses in MOG Antibody–Associated Disease

**DOI:** 10.1002/brb3.70127

**Published:** 2025-03-09

**Authors:** Daniel Lordelo San Martin, Marcos Baruch Portela Filho, Chiara Rocchi, Shahd Hamid, Saif Huda

**Affiliations:** ^1^ NMOSD National Service Walton Centre Foundation Trust Liverpool UK; ^2^ Postgraduate Program in Health Sciences Federal University of Bahia Salvador Brazil; ^3^ Deparment of Life Science State University of Bahia Salvador Brazil

**Keywords:** brain, brainstem/cerebellum, myelin oligodendrocyte glycoprotein antibody‐associated disease, myelitis, optic neuritis, relapse, retrospective cohort, same location

## Abstract

**Background:**

Studies in MS and NMOSD have shown that relapses can frequently occur in the same location as the first attack. Factors associated with this outcome in MOGAD are unclear.

**Objective:**

The objective of this study was to investigate the likelihood of a relapse occurring at the same site in MOGAD.

**Methods:**

This was a UK national cohort study. MOGAD patients with a minimum of one relapse and one year of follow‐up were included. To identify factors associated with relapse location, logistic regression was performed.

**Results:**

An increased risk of a relapse in the same location was observed when the first attack was optic neuritis—for the second attack (OR 12.9, 95% CI 3.31–50.55, *p* = .001) and all subsequent attacks (OR 5.39 95% CI 1.61–18.03, *p* = .006). Conversely, a reduced risk of relapse in the same location was associated with transverse myelitis—for the second attack (OR 0.25, 95% CI 0.07–0.82, *p* = .022) and all subsequent attacks (OR 0.25 95% CI 0.06–0.96, *p* = .045).

**Conclusion:**

In relapsing MOGAD, patients with optic neuritis are at high risk of a new attack in the same location, while those with transverse myelitis are at low risk.

## Graphical abstract

Retrospective cohort from the United Kingdom regarding MOGAD relapsing patients to investigate the new attack pattern. Sixty‐three patients were included and patients with optic neuritis as a first clinical manifestation have a high risk of a new attack in the same location. Conversely, patients with transverse myelitis are less likely to have a relapse in the same topography.

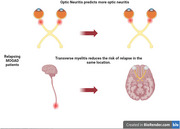

## 1 Introduction

Myelin oligodendrocyte glycoprotein antibody‐associated disease (MOGAD) is an autoimmune, demyelinating disease frequently characterized by attacks of optic neuritis, transverse myelitis, or acute disseminated encephalomyelitis (de Mol et al. 2020). The disease course may be monophasic or relapsing, and new diagnostic criteria have recently been proposed (Banwell et al. 2023), updating the 2018 international consensus (Jarius et al. 2018).

Studies of multiple sclerosis (MS) (Mowry et al. 2009) and neuromyelitis optica spectrum disorder (NMOSD) (Zandona et al. 2014) have shown that relapses often occur in the same location. This is a crucial consideration in MOGAD, where recovery is generally good; however, subsequent attacks in the same location could lead to cumulative disability. Only one prior study from Korea reported an increased risk of relapse in the same location as the initial attack (Hyun et al. 2021). However, these results have not been validated in another population or with application of the 2023 MOGAD diagnostic criteria.

We investigated the probability of relapse occurring in the same of different locations as the inaugural attack in MOGAD in a cohort of UK patients.

## 2 Methods

This is a retrospective cohort. All patients from Northern Ireland, Scotland, and Northern England were seen at the Walton Centre, UK, between July 1, 2013 and September 30, 2023, and we extracted the data since the index attack, which may have occurred prior to the commencement of this study period. Patients fulfilling MOGAD 2023 diagnostic criteria (Banwell et al. 2023) with at least one clinical relapse and 1 year of follow‐up were eligible. MOG‐IgG1 was detected using a live cell‐based assay employing full‐length human MOG (α1 isoform; Oxford Autoimmune Neurology Group) as previously reported (Waters et al. 2015). Clinical data including age at onset, sex, ethnicity, relapses, annualized relapse rate (ARR), disability score as measured by Expanded Disability Status Scale (EDSS), follow‐up length, co‐morbidities, concurrent autoimmune diseases, magnetic resonance image (MRI) results, and death were extracted from case records.

Anatomical sites were categorized into three regions: optic nerve, spinal cord, or brain. If relapses involved multiple locations, each location was included separately in the analysis. Relapses were clinically defined and required the presence of new or worsening neurological symptoms, supported by examination and/or imaging findings, lasting more than 24 h, in the absence of infection, and occurring more than 30 days after a previous attack.

### 2.1 Statistical Analysis

Categorical variables are reported as frequencies and proportions. For normally distributed continuous variables, mean and standard deviations are given; otherwise, median and interquartile range (IQR) are used. Logistic regression was used to analyze if the first central nervous system (CNS) attack region was predictive of a second, third, or all subsequent attack regions. Results are reported as odds ratio (OR) with 95% confidence intervals (95% CI). Variables with a *p*‐value < .1 were included in the multivariable analysis which was adjusted for age at onset, sex, and other potential confounding factors. All analyses were performed using SPSS 21.0.

This study was approved by the Research Ethics Service, NRES Committee London‐Hampstead, Ref. No. 15/LO/1433. All patients provided written informed consent. Guidelines from Strengthening the Reporting of Observational Studies in Epidemiology (STROBE) were followed (von Elm et al. 2007).

## 3 Results

**FIGURE 1:**
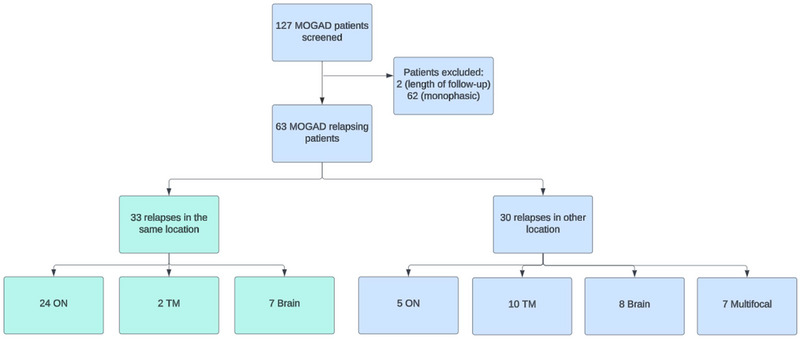
Flow diagram of relapsing MOGAD patients and relapse location. MOGAD, myelin oligodendrocyte glycoprotein antibody‐associated disease; ON, optic neuritis; TM, transverse myelitis.

Sixty‐three patients were included (Figure 1). The median age of onset was 27 (IQR 16–39) years, and 20 patients were less than 18 (32%). Most patients were female (65%) and White (95%) (Table 1). In total, there were 252 clinical attacks. Twenty‐six patients (41%) experienced two attacks, and 37 (59%) experienced three or more attacks. The median disease duration was 9.2 (IQR 5.4–16) years, and the median time between the first and second attacks was 12 (IQR 5–54) months. Index attacks were treated with intravenous methylprednisolone in 29 (46%) patients and plasmapheresis in three (4%) patients. Complete recovery was observed in 26 (41%) patients, partial recovery in 12 (19%), and no recovery in 5 (8%). The remainder of patients were missing this information. Only three patients had an EDSS ≥ 6 at the final follow‐up.

**TABLE 1:** Demographic and clinical aspects of the cohort.

Characteristics	Total
Age at onset, median (IQR)	27 (16–39)
Female, *n* (%)	41 (65)
Ethnicity, *n* (%)	
White	59 (94)
Asian	4 (6)
Time since the first attack until the end of follow‐up in months, median (IQR)	110 (65–192)
First syndrome, *n* (%)	
Optic neuritis	29 (46)
Transverse myelitis	12 (19)
Brain/brainstem	15 (24)
Multifocal onset	7 (11)
Relapses sites, *n* (%)	
Optic neuritis	110
Transverse myelitis	41
Brain/brainstem	24
Multifocal onset	14
First EDSS, median (IQR)	4.0 (3.0–6.5)
Last EDSS, median (IQR)	1.0 (1.0–3.0)
Autoimmune disease, *n* (%)	4 (6)
Annualized relapse rate, median (IQR)	0.3 (0.2–0.6)
Death, *n* (%)	3 (5)

Abbreviations: EDSS, expanded disability scale score; IQR, interquartile range.

### 3.1 Second Relapse Location

**FIGURE 2:**
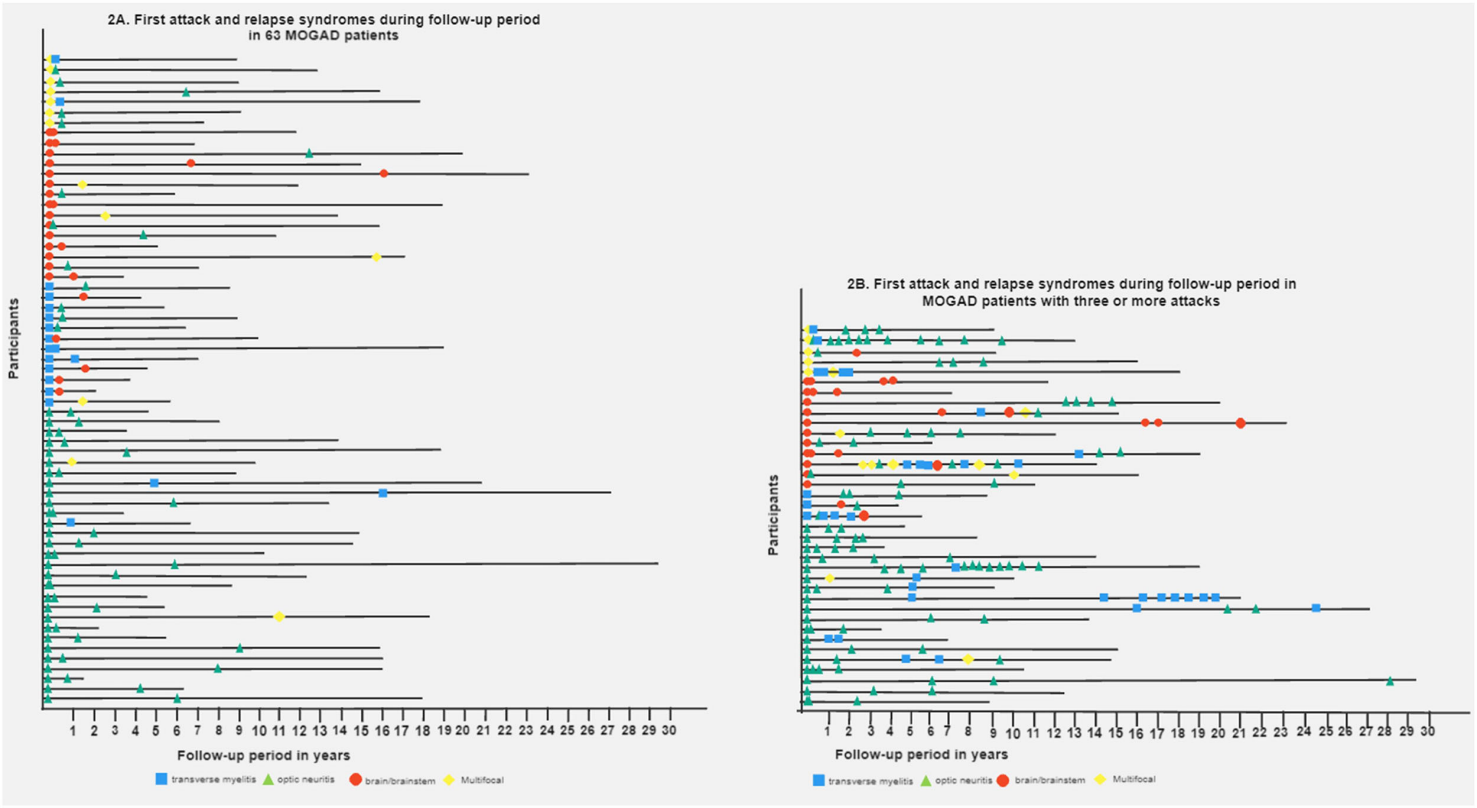
Location of attacks over time in relapsing MOGAD patients. MOGAD; myelin oligodendrocyte glycoprotein antibody‐associated disease.

Figure 2A summarizes the site of attacks over time in relapsing MOGAD patients. In patients with a second attack in the same or different location, there were no differences in age, sex, time between attacks, smoking history, final EDSS, and co‐existent autoimmune disease (data not shown). In the multivariable analysis, optic neuritis was associated with a higher risk of relapse in the same location (OR 12.9, 95% CI 3.31–50.55, *p* = .001). Transverse myelitis showed the opposite effect: relapses in other CNS regions were more likely (OR 0.25, 95% CI 0.07–0.82, *p* = .022). No association with relapse location was observed with brain involvement at onset (Table 2).

**TABLE 2:** Univariable and multivariable logistic regression analysis of attack location in patients with relapsing MOG‐antibody associated disease (adjusted for age and sex).

	Univariable analysis for second attack prediction		Multivariable analysis for second attack prediction		Univariable analysis for third attack prediction	
Variables	OR (95% CI)	*p*‐value	OR (95% IC)	*p*‐value	OR (95% CI)	*p*‐value
Age at onset*	1.01 (0.98–1.05)	.40	1.00 (0.96–1.04)	.74	1.00 (0.96–1.04)	.82
Sex*	1.53 (0.49–4.77)	.46	1.23 (0.28–5.36)	.78	0.35 (0.08–1.47)	.15
First EDSS ≥ 4	0.44 (0.14–1.37)	.16			0.54 (0.13–2.16)	.39
ARR	0.48 (0.08–2.76)	.42			0.68 (0.08–5.82)	.73
ON at first presentation	9.01 (2.67–30.46)	**<.01**	12.9 (3.31–50.55)	**<.01**	1.30 (0.32–5.27)	.71
TM at first presentation	0.25 (0.08–0.81)	**.02**	0.25 (0.07–0.82)	**.02**	0.25 (0.04–1.57)	.26
Brain attack at first presentation	0.80 (0.24–2.60)	.71			1.17 (0.26–5.23)	.83

Abbreviations: ARR, annualized relapse rate; EDSS, expanded disability status scale; CI, confidence interval; MRI, magnetic resonance image; ON, optic neuritis; OR, odds ratio; TM, transverse myelitis. *Multivariable analysis was adjusted for age at onset and sex.

### 3.2 Third and Subsequent Relapse Locations

Figure 2B summarizes the site of attacks over time in MOGAD patients with three or more attacks. There was no association between the disease onset location of the first and second attack and third attack (Table 2). However, when considering all subsequent relapses, optic neuritis was associated with future optic neuritis (OR 5.39 95% CI 1.61–18.03, *p* = .006). Transverse myelitis at onset was again associated with an increased probability of relapse in other CNS locations (OR 0.25 95% CI 0.06–0.96, *p* = .045). Brain involvement was not predictive of relapse in the same or other CNS location.

## 4 Discussion

Although recovery from MOGAD attacks is generally favorable compared to NMOSD, the risk of disability accrual from relapses remains a concern. Data from neurofilament profiles suggest that the first MOGAD attack is typically the most disabling (Mariotto et al. 2021). However, the cumulative impact of subsequent attacks in the same location, depending on the neuronal‐glial reserve, could result in substantial and progressive disability. In this study, we found that patients with MOGAD fulfilling the 2023 diagnostic criteria and with an initial episode of optic neuritis had a significantly increased risk of subsequent attacks involving the optic nerve. Indeed, chronic relapsing inflammatory optic neuropathy (CRION) can be a manifestation of MOGAD, and our data support this finding (Lee et al. 2018). Conversely, patients with transverse myelitis were more likely to experience relapses in other CNS locations. Thus, the clinical phenotype of relapsing transverse myelitis may warrant consideration of alternative diagnoses, particularly if MOG‐IgG results are inconsistent.

A Korean study also reported an increased risk of recurrent optic neuritis in a cohort of MOGAD patients (Hyun et al. 2021). However, unlike our study, they found that myelitis, brain, and brainstem/cerebellum attacks were also likely to recur in the same location. The differences between these studies could be attributed to several factors including selection biases inherent to retrospective tertiary center studies, the inclusion criteria of MOGAD 2023 diagnosed patients in our study, or racial differences between White and Korean population.

The precise mechanisms underlying relapses in the same location in MOGAD remain unclear and have also been described in NMOSD and MS (Mowry et al. 2009; Zandona et al. 2014). A T cell receptor transgenic mouse model, which expresses MOG‐specific T‐cell receptors against the MOG 35–55 peptide, displays spontaneous optic neuritis but not myelitis or other features of experimental autoimmune encephalomyelitis. This is thought to be due to higher MOG expression in the optic nerve compared to the spinal cord, suggesting a potential mechanism for optic neuritis susceptibility (Bettelli et al. 2003). However, it remains unclear whether a similar differential pattern of MOG expression exists in humans. The characteristic and prominent swelling of the optic nerve head and perineural enhancement seen in MOGAD is indicative of blood—optic nerve barrier breakdown. Whether this more pronounced involvement somehow renders the optic nerve susceptible to future attacks is also an area that would be worthwhile investigating further. For instance, in patients in the clinical trial of Inebelizumab in NMOSD, asymptomatic optic nerve enhancement was seen on routine imaging (Cree et al. 2019) and if present in MOGAD could suggest a reduced threshold for entry by peripheral CD4^+^ T‐cells and MOG‐IgG (Corbali and Chitnis 2023).

This was a retrospective observational study precluding the use of randomly assigned groups with similar characteristics. Nonetheless, our study groups did not show significant disparities in general characteristics. The small sample size of brain attacks and limited number of children included where ADEM is prevalent restricted our analysis. Additionally, inadvertent selection bias may be present as patients were seen in a tertiary neurology center, though national (UK) patient referral system may have offset this slightly. A large multicentric prospective study of incident MOGAD in adults and children fulfilling 2023 diagnostic criteria would address these limitations.

In conclusion, our results indicate that MOGAD patients who initially present with optic neuritis are more likely to experience subsequent attacks in the same location, potentially leading to cumulative disability. Conversely, patients presenting with transverse myelitis are less likely to have relapses in the same location, suggesting different patterns of disease progression. These findings underscore the need for tailored monitoring and management strategies based on the initial presentation of MOGAD to mitigate long‐term disability.

## Author Contributions

**Daniel Lordelo San Martin**: conceptualization, investigation, funding acquisition, writing–original draft, methodology, validation, visualization, writing–review and editing, software, formal analysis, project administration, data curation, resources. **Marcos Baruch Portela Filho**: formal analysis, resources, data curation, visualization, investigation, conceptualization. **Chiara Rocchi**: conceptualization, investigation, methodology, validation, visualization. **Shahd Hamid**: conceptualization, investigation, methodology, validation, visualization, writing–review and editing, supervision. **Saif Huda**: supervision, writing–review and editing, writing–original draft, investigation, conceptualization, methodology, validation, visualization, project administration.

## Conflicts of Interest

The authors declare no conflicts of interest.

### Peer Review

The peer review history for this article is available at https://publons.com/publon/10.1002/brb3.70127.

## Data Availability

The data that support the findings of this study are available on request from the corresponding author. The data are not publicly available due to privacy or ethical restrictions.
